# An Explanation

**Published:** 1888-11

**Authors:** 


### AN EXPLANATION.

Some of our readers who take the *Dental Cosmos* may have thought strange that three of the papers of the Pennsylvania State Society meeting should appear in full in that journal, when we were elected official reporter for the society for the current year. It was especially irritating, in that we had made one of them, that of Dr. Kirk, a leader in our original communications.

We are satisfied that it was an oversight, and accept as the *amende honorable* the explanation found in a short editorial in the October number of the *Cosmos;* as follows:

"Owing to a misunderstanding, some of the papers read at the Pennsylvania State Dental Society, which, by virtue of a prior arrangement belonged to the INDEPENDENT PRACTITIONER, were printed in full in the *Dental Cosmos* for September. It is scarcely necessary to say that the *Dental Cosmos* would not wilfully violate the courtesies of honorable journalism or the rights of another journal. It does not, except by inadvertence, print papers that are not intended for its columns, much less those intended for others."

We do not intend to print any articles under the heading of original communications which have appeared in any other journal; and not only that, but we intend, as far as possible, to have the exclusive control of the proceedings of the societies for which we report, except, of course, if other journals desire abstracts we shall not object. We believe that there is room for us all to follow out different lines, for the benefit of our readers and the journals also.

